# The relationship between complex PTSD and dissociation: A longitudinal study

**DOI:** 10.1192/j.eurpsy.2025.1951

**Published:** 2025-08-26

**Authors:** H. W. Fung, C. M. Li, C. H. O. Huang, M. Y. C. Wong, T. Y. N. Tsui, A. K. C. Chau

**Affiliations:** 1 School of Nursing, The Hong Kong Polytechnic University, Kowloon, Hong Kong; 2 Department of Psychiatry, Yale University School of Medicine, New Haven, United States; 3 Department of Social Work and Social Administration, The University of Hong Kong, Pokfulam; 4 Department of Health and Physical Education, The Education University of Hong Kong, Taipo, Hong Kong; 5 School of Psychology, Australian Catholic University, Melbourne, Australia; 6 Department of Psychiatry, the University of Hong Kong, Pokfulam, Hong Kong

## Abstract

**Introduction:**

Complex post-traumatic stress disorder (C-PTSD) is closely associated with dissociative symptoms. Both of which are common responses to trauma and stress. Yet, not all individuals with C-PTSD experience high levels of dissociation. Currently, little is known about the bidirectional relationship between C-PTSD and dissociative symptoms.

**Objectives:**

This study aimed to examine whether C-PTSD and dissociative symptoms would predict each other over time.

**Methods:**

A total of 340 participants (M_age_=21.04 years; SD=2.00; 83.8% female) from Hong Kong and Taiwan completed the Multiscale Dissociation Inventory (MDI) and the International Trauma Questionnaire (ITQ) at two separate time points (M days apart = 129.4 days; SD = 7.91). Hierarchical multiple regression analyses were conducted to examine the relationship between C-PTSD and dissociative symptoms.

**Results:**

The analyses controlled for age, gender, education level, trauma exposure, and baseline severity of the dependent variables. Results indicated that when the MDI subscales were added into the model, baseline emotional constriction significantly predicted subsequent C-PTSD symptoms (i.e., total ITQ scores) (β=.126, p=.008), and significantly improved the model’s explanatory power (R²=.67, ΔR²=.029, ΔF = 4.772, p < .001). Nevertheless, when the same analysis was conducted, none of the six C-PTSD symptom clusters at baseline predicted the total MDI scores at follow-up (ΔF = 1.000, p = .425).

**Conclusions:**

The study findings suggested that dissociative symptoms in general, and emotional constriction in particular, predicted subsequent levels of C-PTSD symptoms, while C-PTSD symptoms did not predict subsequent levels of dissociation. These results highlighted that proactive management of dissociative symptoms might be an important part of the treatment of C-PTSD. This study provided a foundation for future research to investigate the underlying mechanisms by which emotional constriction influences C-PTSD severity. Future research should also evaluate dissociation-focused interventions for people with C-PTSD.

**Disclosure of Interest:**

None Declared

